# Therapeutic Use of Psilocybin in Depression: a Systematic Review of Clinical Evidence

**DOI:** 10.1017/neu.2025.10039

**Published:** 2025-09-03

**Authors:** Filipe Reis Teodoro Andrade, Tobias Buchborn, Gabriel Thalheimer, Marcus W. Meinhardt, Samia Joca, Rosa Maria Martins de Almeida

**Affiliations:** 1 Department of Psychopharmacology, Central Institute of Mental Health (ZI), Mannheim, Germany; 2 Department of Developmental and Personality Psychology, Laboratory of Experimental Psychology, Neuroscience and Behavior (LPNeC), Universidade Federal of Rio Grande do Sul (UFRGS), Porto Alegre, Brazil; 3 Department of Biomedicine - Forskning og uddannelse, Vest, Aarhus University, Aarhus, Denmark

**Keywords:** Depressive Disorder, Psilocybin, Psychedelics, Systematic Review, 5HT2a receptor

## Abstract

**Background:**

Major depressive disorder (MDD) is a significant public health concern, and current treatments often have limitations in effectiveness and adherence. Psilocybin, a psychedelic compound found in certain mushrooms, is being explored as a potential treatment for depression. It primarily acts through the serotonin 5-HT2A receptor but interacts with 5-HT1A and 5-HT2C receptors. Its precise mechanisms remain under investigation.

**Objectives:**

(1) To consolidate evidence on psilocybin’s efficacy and safety for depression and the role of 5HT2a, (2) to identify limitations in the literature, and (3) to highlight areas needing further research.

**Methods:**

This systematic review follows PRISMA guidelines and analyses 22 studies, including randomised controlled trials (RCTs) and open-label studies. The studies cover various populations, including individuals with treatment-resistant depression, different dosing regimens, and adjunctive therapies.

**Results:**

Psilocybin therapy shows substantial and rapid antidepressant effects, often after one or two sessions with psychological support. Improvements are sustained for weeks or months in many cases. Psilocybin is generally well-tolerated, with mild adverse effects such as anxiety during administration and transient headaches, which are manageable in controlled settings.

**Conclusions:**

Psilocybin demonstrates promise as a novel treatment for depression, especially for individuals unresponsive to conventional antidepressants. Further research is needed to refine dosing, explore long-term effects, and understand its mechanisms of action.

## Graphical abstract

**Figure:**
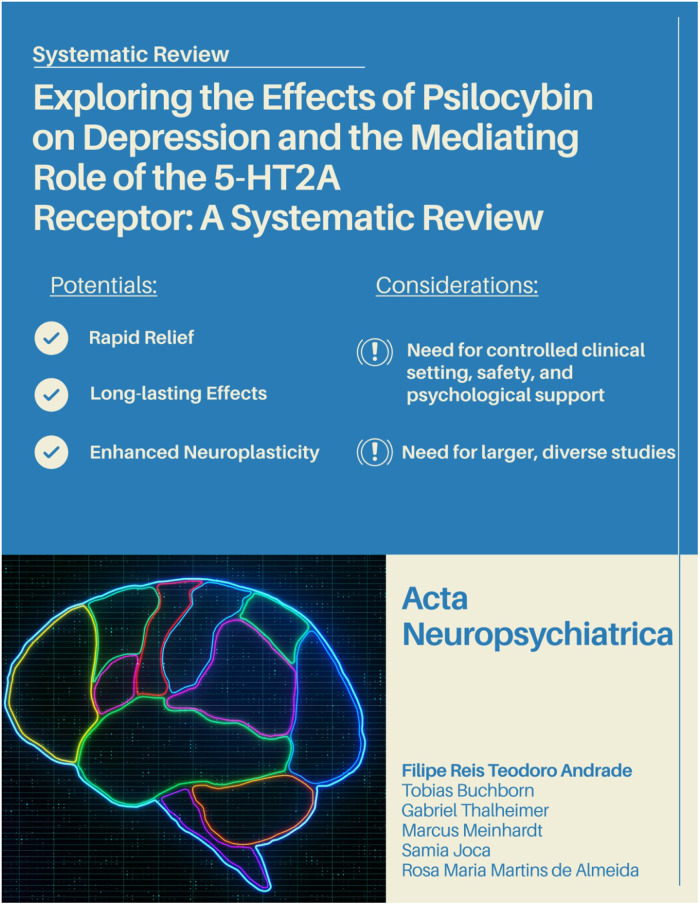

Summations

- Rapid and Sustained Antidepressant Effects: Psilocybin therapy has demonstrated rapid and substantial reductions in depressive symptoms, often within a single or few doses, with effects sustained for weeks to months.
- 5-HT2A Receptor and Neuroplasticity: The antidepressant effects of psilocybin are primarily mediated through 5-HT2A receptor activation, promoting synaptic plasticity and emotional processing, differentiating it from traditional antidepressants.
- Controlled Use and Safety Profile: While generally well-tolerated, psilocybin therapy requires structured clinical settings with psychological support.

Considerations

- Small Sample Sizes and Short Follow-Ups: Many studies involve small cohorts and short observation periods, limiting the generalisability of long-term effects and relapse rates.
- Lack of Standardised Dosing and Protocols: Variability in psilocybin doses, session frequency, and psychological support structures complicates direct comparisons and clinical applicability.
- Limited Diversity in Study Populations: Most studies focus on Western populations, with minimal representation of diverse ethnic, cultural, and socioeconomic backgrounds, affecting external validity.

Highlights

- Psilocybin exhibits rapid and sustained antidepressant effects, often after one or two sessions, with improvements lasting weeks to months;
- Its therapeutic mechanism primarily involves activation of the 5-HT2A receptor, which promotes neuroplasticity and emotional processing essential for alleviating depression;
- Current research faces limitations such as small sample sizes, short follow-up periods, lack of standardised protocols, and limited diversity, underscoring the need for further comprehensive studies.

## Introduction

Depression is a chronic mental disorder that affects millions globally, and according to the World Health Organization (WHO, 2019), over 350 million people worldwide suffer from this condition. Approximately 40.27 million individuals in Europe are affected, constituting 4.3% of the population. At the same time, in the United States, the National Institute of Mental Health (NIMH, 2023) reported that 8.4% of adults experienced at least one major depressive episode in 2021. Women are more likely than men to experience depression, and it is most commonly observed among young and elderly populations (Salk *et al*., 2017; Kirkbride *et al.,* 2023; Wang *et al*., 2024). Symptoms of depression can include persistent sadness, loss of interest in previously enjoyable activities, reduced attention, pessimism about the future, and feelings of guilt or unworthiness. Physical symptoms such as chronic pain, fatigue, sleep disturbance, appetite changes, and weight fluctuations are also common. Patients can be classified as having mild, moderate, or severe depression, with psychological therapy and pharmacotherapy being the primary treatment modalities (Fergusson *et al*., 2005; Klein *et al*., 2009; Kirkbride *et al*., 2023; Bacigalupe *et al*., 2024).

The most commonly prescribed medications for depression fall into five categories: serotonin reuptake inhibitors (SRIs), serotonin and norepinephrine reuptake inhibitors (SNRIs), tricyclic antidepressants, monoamine oxidase inhibitors (MAOIs), and atypical antidepressants (Boschloo *et al*., 2023). These medications primarily act as monoamine modulators and have demonstrated efficacy in mood disorder treatment. Medicinal chemistry and pharmacological data underscore the importance of serotonin (5-HT) and the 5-HT2A receptor in the pathophysiology of depression, with recent findings highlighting the rapid antidepressant effects of ligands targeting this receptor (Hieronymus *et al*., 2019; Kaiser *et al*., 2021; Boschloo *et al*., 2023).

While traditional pharmacotherapy remains a cornerstone of treatment, alternative approaches, including psychedelic medicine, have garnered significant research interest. Psychedelics may influence depression through mechanisms tied to neuroplasticity and emotional processing. For instance, studies have shown that the intensity and emotional resonance of a psychedelic experience can positively affect depressive symptoms (Griffiths *et al*., 2006; Carhart-Harris *et al*., 2016; Carhart-Harris and Goodwin, 2017; Roseman *et al*., 2018).

These effects are thought to stem from the ability of psychedelics to engage specific receptors and induce structural and functional neuroplasticity. Advances in electrophysiological methods to study brain plasticity, such as those described by De Vos *et al.* (2022), have provided more profound insights into how psychedelics induce these neuroplastic changes, bridging the gap between preclinical and clinical research.

Amid these findings, the continued prevalence of depression emphasises the need for innovative treatment approaches. In 2019, WHO reported that approximately 970 million people worldwide, or one in eight, suffered from mental disorders, with depression and anxiety being the most common World Health Organization (WHO). These statistics underline the urgency for expanding therapeutic options and advancing our understanding of this complex condition, offering hope for better outcomes for sufferers worldwide.

The 5-HT2A receptor is a crucial mediator in the pharmacodynamics of psychedelic drugs, primarily facilitating their psychoactive effects through activation of serotonin 5-HT2A receptors predominantly located in the cortical regions of the brain. These receptors are densely expressed in the prefrontal cortex associated with higher-order cognitive functions. Their activation by psychedelics such as psilocybin, LSD, and DMT leads to profound alterations in perception, cognition, and emotion, which are hallmark features of the psychedelic experience (Cameron *et al*., 2023; Pędzich *et al.,* 2022; Nichols, 2016).

Upon activation of the 5-HT2A receptors, psychedelics trigger a cascade of intracellular signalling pathways, particularly involving the phospholipase C (PLC) pathway. This pathway subsequently increases the production of inositol trisphosphate (IP3) and diacylglycerol (DAG), leading to the release of calcium ions from intracellular stores (Raote and Bhattacharya, 2020).

The activation of 5-HT2A receptors by psychedelics modulates the excitability of cortical neurons and influences synaptic plasticity. These mechanisms underpin the profound changes in consciousness and perception these substances induce (Preller and Vollenweider, 2019).

Moreover, the activation of 5-HT2A receptors influences glutamatergic neurotransmission by enhancing glutamate release in the prefrontal cortex. This glutamate release, in turn, affects the activity of other neurotransmitter systems, such as dopamine and GABA, contributing to the broad-spectrum effects on neural circuits that underpin the psychedelic state (Vollenweider and Kometer, 2010). The intricate interplay between these neurotransmitter systems and the 5-HT2A receptor is still being explored to delineate the full scope of psychedelic-induced neuroplasticity and its potential therapeutic applications in treating psychiatric disorders (Wing *et al.,* 1990; Pędzich *et al*., 2022).

One of the most significant effects of the psychedelic experience triggered by the activation of the 5-HT2A receptors is the profound changes in perception and thought processes. This may include visual hallucinations, altered sense of time, and deep introspective or existential thoughts. These effects have been used traditionally in various cultures and studied more recently for the therapeutic potential for mental health conditions such as depression, anxiety, and PTSD (Hoskins *et al*., 2015; Steenkamp *et al.,* 2015).

To sum up, the 5-HT2A receptor’s role in psychedelics is critical to the psychotropic effects these substances produce, significantly impacting perception, cognition, and emotional states. Growing evidence suggests that psilocybin in low doses can help patients with depression symptoms (Gasser *et al*., 2014; Sessa, 2015; Carhart-Harris *et al*., 2018). The side effects are very low, and the systematic reviews have found that psychedelic drugs, when associated with safe use in a safe place, could help in the well-being of patients with depression or cancer terminal (De Vos *et al*., 2022; Amsterdam *et al*., 2022).

Existing reviews on psilocybin and depression often lack the methodological rigour needed to minimise bias and subjectivity. Furthermore, many do not adhere to the Preferred Reporting Items for Systematic Reviews and Meta-Analyses (PRISMA) guidelines or pre-registration protocols, which ensure transparency, reproducibility, and methodological consistency. This review seeks to address these gaps by employing a systematic approach guided by PRISMA and pre-registering the protocol in PROSPERO. By adhering to these standards, this review aims to provide a comprehensive and unbiased synthesis of the current evidence, critically evaluating the therapeutic potential of psilocybin in depression treatment.

In doing so, this review serves several objectives: (1) to consolidate existing evidence on the efficacy and safety of psilocybin for depression and the role of 5HT2a, (2) to identify limitations and inconsistencies in the current literature, and (3) to highlight areas requiring further investigation. By addressing these gaps, this review aims to enhance the understanding of psilocybin’s role in depression management and inform future research and clinical practice.

## Materials and methods

A systematic review was carried out according to the Preferred Reporting Items for Systematic Reviews and Meta-Analyses (PRISMA) statement guidelines. The research question and search strategy were formulated using the PICO (Population, Intervention, Comparator, and Outcome) framework, which can be found in Table 3 of the supplementary material. The protocol was registered with PROSPERO under the CRD42023457537 registration number.

### Search methods

Literature searches were conducted using five online databases: Embase, Scopus, PsycINFO, PubMed, and Web of Science to identify studies with relevant information. We searched for studies in any language between 2018 and 2023 along with an additional two studies from 2025, which refer to the research on the effects of psilocybin on the 5HT2a receptor in a depression model. The terms used were: [(psilocybin[MeSH Terms]) OR (psilocybin) OR (psilocybin) OR (psilocybin) OR (psiloc*)] AND [(depressive disorder [AND] (5HT2a)] psilocybin OR psilocybin OR psilocybine OR psilocybin OR psiloc* AND depressive AND disorder AND 5ht2a))’. No restrictions were applied on country, gender, or race/ethnicity.

### Search strategy

Two authors (FRTA and GT) independently identified most of the double-blind RCTs published before 1 November 2023. In April 2025 (FRTA, GT, and RMMA), two more studies were added that examined the efficacy and safety of psilocybin in the treatment of MDD.

### Inclusion and exclusion criteria

Original articles reporting the effects of psilocybin or other psychedelic drugs on depression were included. Studies focused on other mental health disorders, animal models, editorials, opinion pieces, conference abstracts, case reports, systematic reviews, meta-analyses, and articles not published in English were excluded. Also, studies involving healthy participants (Holze *et al*., 2022; Madsen *et al.,* 2020; Barret *et al.,* 2018; Carbonaro *et al.*, 2018; Ley *et al*, 2023) were included, with a justified rationale outlined below.

Including studies conducted with healthy participants in this systematic review was justified for several key reasons. First, these studies offer valuable mechanistic insights into how psilocybin modulates brain function, mainly through serotonergic pathways directly implicated in depression. Understanding these mechanisms is essential for interpreting psilocybin’s potential therapeutic effects. Second, they provide critical safety and tolerability data, which is a necessary foundation for designing and conducting clinical trials in vulnerable populations such as those with depression. Third, studies in healthy participants help determine the dose–response relationship and characterise subjective effects, such as altered states of consciousness, which are hypothesised to mediate therapeutic outcomes in clinical populations.

While studies with healthy participants do not directly measure antidepressant efficacy, they play a pivotal role in bridging preclinical research (e.g., animal models) and clinical trials. By providing data on pharmacokinetics, subjective experiences, and safety profiles, these studies contribute to developing appropriate therapeutic protocols. Therefore, including such studies broadens the scope and depth of this review by contextualising findings from clinical trials, enhancing its overall comprehensiveness.

### Data extraction

Two authors (FRTA and GT) independently extracted the following data from each included RCT using a tailored form: author, year of publication, methods, instruments, country, clinical trials, aims, number of sessions, type of drugs, decreased depression, related side effects, sample, and conclusions. Any discrepancies were resolved by discussion and adjudication through a senior author. If the study data needed to be clarified, the first/corresponding authors were contacted by email to obtain further information.

### Assessment of study quality

To ensure methodological rigour, three authors (FRTA, GT, and RMMA) assessed study quality independently using the Newcastle–Ottawa Scale (NOS) and the Jadad scale. The NOS evaluates non-randomised studies across three key domains: selection, comparability, and outcome assessment (Wells *et al*., 2000). The Jadad scale was applied for randomised controlled trials, with a score of ≥ 3 indicating high-quality studies (Jadad *et al*., 1996). Additionally, the overall quality of primary and secondary outcomes was evaluated using the Grading of Recommendations, Assessment, Development, and Evaluation (GRADE) system, providing a structured framework to rate the certainty of evidence. All assessments were performed independently by FRTA and RMMA, with discrepancies resolved through discussion.

## Results

### Search results

**Figure 1.**
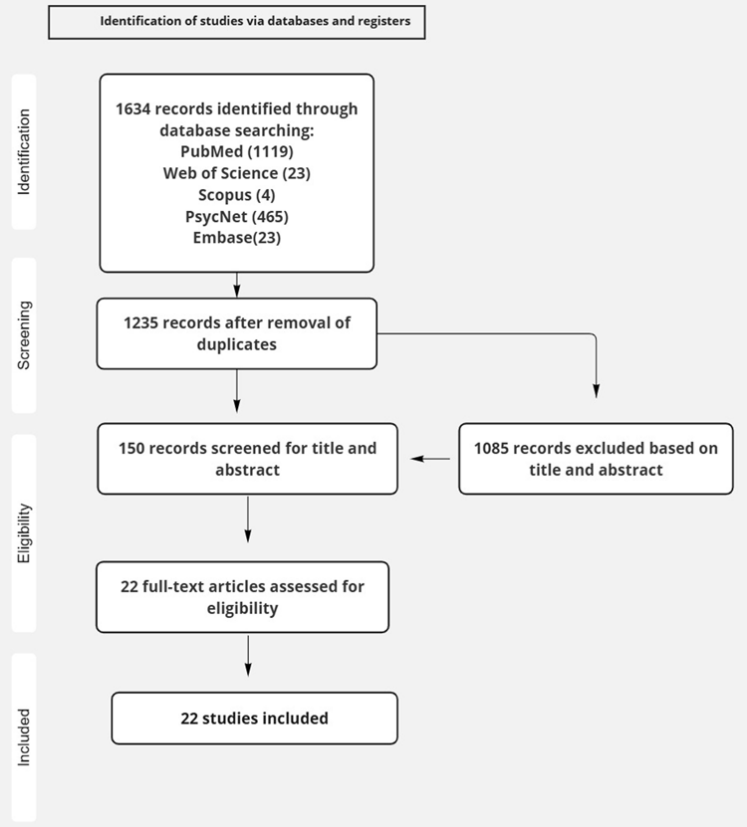
Flow char for selection of studies.

A comprehensive search identified 1,634 articles (Fig. 1), of which 105 studies met the inclusion criteria. 150 studies were excluded for not meeting the eligibility requirements. Exclusion criteria included review articles or meta-analyses, non-English language publications, studies using animal models, or investigations involving other psychiatric conditions such as addiction or bipolar disorder. Ultimately, 22 studies were included in the final analysis.

### Considerations regarding included studies

Some included studies (Carhart-Harris *et al*., 2021; Barba *et al*., 2022; Murphy *et al*., 2022; Goodwin *et al*., 2022) were derived from the same randomised clinical trials, sharing participant samples and protocols. However, these studies report on different outcomes, sub-analyses, or follow-up measures. In accordance with our predefined criteria, these studies were retained because each contributes unique and complementary insights into psilocybin’s therapeutic profile.

### Characteristics of the included studies

The 22 included studies primarily focused on psilocybin’s efficacy in treating major depressive disorder (MDD) and treatment-resistant depression (TRD). These studies employed robust methodologies, particularly randomised clinical trials (RCTs), to examine psilocybin’s therapeutic potential. Sample characteristics for each study are provided in Table 1, and additional methodological details are summarised in Tables 2–3 (Supplementary Material).

Examples include Skosnik *et al*. (2023), who used a double-blind, placebo-controlled, within-subject design to assess EEG correlates of neural plasticity in MDD, and Ley *et al*. (2023), who employed a double-blind, placebo-controlled crossover design. Phase II trials, such as Goodwin *et al*. (2023), further evaluated safety and efficacy.

**Table 1.S:** Selected Studies.

Author (year)	Country	Methods	Sample	Aims	Number of Sessions	Instruments	Type of drug	Related side effects	Conclusion
Aaronson *et al.,* 2025	United States	12-week, open-label clinical trial involving a single 25 mg dose of synthetic psilocybin with preparatory and integration psychotherapy.	*N* = 12 (6 male, 6 female); Mean age = 40.6 years Ethnicity = 100% White.	To assess the safety and antidepressant efficacy of a single dose of psilocybin in individuals with severe treatment-resistant depression (TRD)	3 preparatory therapy sessions; 1 psilocybin dosing session (8–9 hours); 3 integration sessions post-dosing; Regular follow-ups at weeks 1, 2, 3, 6, 9, and 12.	Montgomery-Åsberg Depression Rating Scale (MADRS); Quick Inventory of Depressive Symptoms – Self-Rated (QIDS-SR-16); General Anxiety Disorder-7 (GAD-7); Quality of Life Enjoyment and Satisfaction Questionnaire (QLES-Q-SF); Work and Social Adjustment Scale (WSAS); 5D-Altered States of Consciousness Scale (5D-ASC); Challenging Experience Questionnaire (CEQ); Mystical Experience Questionnaire (MEQ).	Synthetic psilocybin (25 mg, COMP360 formulation by COMPASS Pathways)	Mild headache (16.7%); Moderate insomnia (2 patients); Moderate worsening of depression in 1 patient; Psychomotor agitation (1 case); No serious adverse events reported.	Supports the safety and potential efficacy of psilocybin for severe TRD, with significant symptom improvement observed in most participants.
Erritzoe *et al.,* 2024	United Kingdom	6-month follow-up of a phase 2, double-blind, randomised controlled trial	*N* = 59 (29 male, 20 female) Mean age = 41 years Ethnicity = 52 (85%) participants were White.	To compare the long-term (6 months) effects of psilocybin therapy versus escitalopram on depression severity, social functioning, psychological connectedness, and meaning in life, and to assess their sustainability after intensive treatment.	Psilocybin group: 2 doses administered approximately 3 weeks apart, with around 20 hours of in-person psychological support across the treatment. Escitalopram group: 6-week course of daily medication (‘escitalopram’ at 10 mg and 20 mg) with psychological support.	16-item Quick Inventory of Depressive Symptomatology – Self-Report (QIDS-SR-16); Work and Social Adjustment Scale (WSAS); Watts’ Connectedness Scale (WCS); Meaning in Life Questionnaire (MLQ); Flourishing Scale (FS)	Psilocybin (1 mg) Escitalopram (10 mg, then 20 mg)	Side effects not detailed	Both treatments resulted in sustained reductions in depression severity over six months. Psilocybin therapy was associated with greater improvements in social functioning, connectedness, and meaning in life, beyond symptom reduction.
Husain *et al*., 2023	Canada	Double-Blind Proof-of-Concept Randomised Controlled Trial	*N* = 60 Mean Age = not assessed. Ethnicity = not assessed.	To block the psychedelic effects of psilocybin and provide data on its antidepressant effect.	Not specify the number of days, but is 12 hours of psychotherapy in each session.	Montgomery–Åsberg Depression Rating Scale (MADRS), 5-Dimensional Altered States of Consciousness Rating Scale (5D-ASC), among others	Psilocybin (25 mg) and Risperidone (1 mg)	Side effects associated with the combination of psilocybin and risperidone are examined	The research may increase the acceptability and access to psilocybin for TRD without needing psychedelic experiences
Skosnik *et al*., 2023	United States Of America	Double-blind, placebo-controlled within-subject study	*N* = 19 Mean Age = not assessed. Ethnicity = not assessed.	Investigate the effects of psilocybin on EEG correlates of neuroplasticity and its relationship with depression symptoms.	Two (placebo followed by psilocybin)	Electroencephalography (EEG), GRID Hamilton Rating Scale for Depression-17 (GRID-HAM-D-17), and MADRS)	Psilocybin (0.3 mg/kg) and placebo	Not specifically mentioned	Psilocybin increased auditory evoked theta power two weeks post-administration, correlating with improvements in depression symptoms, indicating potential neuroplastic changes in the brain.
Goodwin *et al*., 2023	22 sites across 7 countries in Europe and North America	Phase 2, multicenter, international double-blind trial	*N* = 233 (52% females) Mean age = 39.8 years Ethnicity = 92% were White.	Evaluate the efficacy of single-dose psilocybin in treatment-resistant depression.	Single dose	Various, including QIDS-SR-16, PANAS, GAD-7, SDS, WSAS, EQ-5D-3L, DSST	Psilocybin (25 mg, 10 mg, and 1 mg doses)	Adverse events in 66 –84% of participants across doses	Single-dose psilocybin showed benefits in reducing depression and anxiety symptoms, and in improving functioning and quality of life in treatment-resistant depression
Ley *et al*., 2023*	Switzerland	Double-blind placebo-controlled crossover design with four experimental test sessions	*N* = 32 (16 men and 16 women) Mean age = 29 Ethnicity = not assessed.	Compare the acute effects of mescaline, LSD, and psilocybin.	Four sessions (300 mg or 500 mg mescaline, 100 µg LSD, 20 mg psilocybin, and placebo)	Adjective Mood Rating Scale (AMRS), and the 5 Dimensions of Altered States of Consciousness (5D-ASC) scale.	Mescaline hydrochloride (99.3% purity), LSD base (> 99% purity), and psilocybin	Mescaline, LSD, and psilocybin were found to be tolerable when used at psychoactive-equivalent doses	No evidence of qualitative differences in altered states of consciousness induced by equally strong doses of mescaline, LSD, and psilocybin. Differences in pharmacological profiles do not translate into relevant differences in the subjective experience.
Weiss *et al*., 2023	United Kingdom	Phase 2 double-blind randomised active comparator-controlled trial	*N* = 59 (29 male, 20 female) Mean age = 41 years Ethnicity = 52 (85%) participants were White.	To examine changes in personality in relation to PT and ET.	Core 6-week trial period	Big Five Inventory, Big Five Aspects Scale, Modified-Tellegen Absorption Scale, Barratt Impulsivity Scale-Brief	Psilocybin and Escitalopram	Side effects not detailed	Both PT and ET resulted in personality changes consistent with improved mental health. No significant between-group differences, except for a trend in absorption changes favouring PT.
Goodwin *et al*., 2023	International (Ireland and the United States)	Phase II exploratory fixed-dose open-label study	*N* = 19 Mean Age = not assessed. Ethnicity = not assessed.	To explore the safety, tolerability, and efficacy of synthetic psilocybin (COMP360) as an adjunct to selective serotonin reuptake inhibitors (SSRIs) in treatment-resistant depression (TRD) patients.	Single administration session, followed by three weeks of follow-up.	Montgomery-Åsberg Depression Rating Scale (MADRS), Clinical Global Impression–Severity (CGI-S), Generalised Anxiety Disorder 7-item (GAD-7).	Synthetic psilocybin (COMP360) alongside SSRIs.	Mostly mild, including headaches and transient blood pressure increase; no serious treatment-emergent adverse events reported.	Psilocybin, when used alongside SSRIs, showed a favourable safety profile and potential efficacy in reducing symptoms of TRD. Further large-scale trials are recommended to confirm these findings.
Raison, *et al*., 2023.	Conducted in the United States.	Randomised, placebo-controlled clinical trial.	*N* = 104 (52 women) Mean age = 41.1 years Ethnicity = not assessed.	To evaluate the antidepressant effects and safety of a single dose of psilocybin compared to niacin in patients with Major Depressive Disorder (MDD).	One dosing session with follow-ups.	Montgomery-Åsberg Depression Rating Scale (MADRS), Sheehan Disability Scale (SDS).	Synthetic psilocybin (25 mg) and niacin as a placebo.	Headaches, nausea, and visual perceptual effects.	Psilocybin treatment led to a statistically significant reduction in depressive symptoms compared to niacin, with rapid onset and sustained effects over six weeks.
Sloshower *et al*., 2023	United States Of America	Placebo-controlled, within-subject, fixed-order study	*N* = 19 Mean age= not assessed. Ethnicity = not assessed.	To assess the efficacy of psilocybin-assisted therapy in treating major depressive disorder.	2 dosing sessions, 8 psychotherapy sessions.	GRID-Hamilton Depression Rating Scale, Quick Inventory of Depressive Symptomatology–Self-Report	Psilocybin (0.3 mg/kg) and placebo (microcrystalline cellulose)	Mild to moderate transient headache, anxiety, and dysphoria; no severe adverse events.	Psilocybin-assisted therapy showed potential benefits in treating major depression, with significant improvements in depression and anxiety scores and quality of life.
von Rotza *et al*., 2023	Switzerland	Randomised, double-blind, placebo-controlled trial	*N* = 52 Mean age = not assessed. Ethnicity = not assessed.	Assess the effectiveness of a single moderate dose of psilocybin in treating MDD	One session, with follow-up assessments	Montgomery-Åsberg Depression Rating Scale (MADRS), Beck Depression Inventory (BDI)	Psilocybin (0.215 mg/kg body weight) and placebo	Headaches, nausea, no severe adverse events	Psilocybin significantly reduced depressive symptoms compared to placebo, with effects lasting for at least two weeks. The treatment was well-tolerated with mild side effects.
Holze *et al*., 2022 *	Switzerland	Double-blind, randomised, placebo-controlled crossover design	*N* = 28 (14 men, 14 women). Mean age = 35 years Ethnicity = not assessed.	Compare acute subjective, autonomic, and endocrine effects of LSD and psilocybin	5 sessions per participant	Visual Analogue Scales (VASs) Adjective Mood Rating Scale (AMRS) 5D-ASC scale States of Consciousness Questionnaire Blood pressure, heart rate, and body temperature measurements Pupil size assessment List of Complaints (LC) Plasma concentrations (cortisol, prolactin, oxytocin, BDNF)	LSD (100 and 200 µg), Psilocybin (15 and 30 mg)	Increased heart rate, blood pressure, body temperature, pupil size, and adverse events	LSD and psilocybin produced comparable subjective and autonomic effects, with differences in duration.
Barba *et al*., 2022	United Kingdom	Randomised clinical trial	*N* = 59 (29 male, 20 female) Mean age = 41 years Ethnicity = 52 (85%) participants were White.	Compare the effects of psilocybin and escitalopram on rumination and thought suppression in major depressive disorder	Psilocybin: 2 sessions; Escitalopram: 6 weeks of treatment	Ruminative Response Scale (RRS), White Bear Suppression Inventory (WBSI), Quick Inventory of Depressive Symptomatology Self-Report (QIDS-SR-16)	Psilocybin, Escitalopram	The overall incidence of adverse events was similar between the psilocybin and escitalopram groups, and no serious adverse events occurred. It is noted that the side-effect profile of psilocybin was less diverse than that of escitalopram and was superior in certain domains, including anxiety, dry mouth, sexual dysfunction, and emotional function.	Psilocybin showed greater impact on reducing rumination and thought suppression compared to escitalopram in the treatment of depression.
Gukasyan *et al*., 2022	United States Of America	Randomised waiting-list controlled study	*N* = 24 (67% were female) Mean age = 39.8 years Ethnicity = 22(92%) were Caucasian, one identified as Black and another as Asian.	To examine the efficacy and safety of psilocybin through 12 months in participants with moderate to severe MDD who received psilocybin.	Two doses of psilocybin.	GRID-Hamilton Depression Rating Scale (GRID-HAMD), Quick Inventory of Depressive Symptoms (QIDS), Beck Depression Inventory II (BDI-II)	Psilocybin	No serious adverse events, low suicidal ideation, no instances of self-injurious behaviour, no reported use of psilocybin or other psychedelics outside of the study, no participant met criteria for HPPD.	Psilocybin-assisted therapy shows substantial antidepressant effects that may be durable at least through 12 months following acute intervention in some patients. 75% of participants met the criteria for clinical response (defined as *a* ≥ 50% reduction in GRID-HAMD scores), and 58% met the criteria for remission (GRID-HAMD ≤7). These results were accompanied by a large effect size (Cohen’s *d* = 2.4), indicating a sustained and meaningful antidepressant effect.
Daws *et al*., 2022.	United States Of America and United Kingdom	Two clinical trials: an open-label trial and a double-blind randomised controlled trial (DB-RCT).	Open-label: *N* = 16 (4 females) Mean age = 42.75 years. Ethnicity = not assessed. DB-RCT: N1 = 22 (6 females) Mean age = 40.9 years N2 = 21 (8 females) Mean age = 44.5 years . Ethnicity = not assessed.	To assess the antidepressant potential of psilocybin and its effects on brain function.	Open-label (2 sessions), DB-RCT (2 sessions of 25 mg psilocybin or 1 mg psilocybin with escitalopram).	Functional magnetic resonance imaging (fMRI).	Psilocybin and escitalopram.	Not related	Psilocybin therapy resulted in decreased brain network modularity, indicating increased global brain network integration. This change correlated with improvements in depression symptoms, suggesting a potential mechanism for the antidepressant effects of psilocybin.
Goodwin *et al*., 2022	22 sites across 7 countries in Europe and North America	Random assignment of adults with treatment-resistant depression to receive a single dose of synthetic psilocybin (25 mg, 10 mg, or 1 mg) with psychological support.	*N* = 233 (52% females) Mean age = 39.8 years Ethnicity = 92% were White	To determine the efficacy and safety of a single dose of psilocybin in treating treatment-resistant depression.	One administration session, with follow-up over 12 weeks.	Montgomery–Åsberg Depression Rating Scale (MADRS)	Synthetic formulation of psilocybin (25 mg, 10 mg, or 1 mg)	Headache, nausea, dizziness, and in some cases, suicidal ideation or self-injury.	Psilocybin (25 mg) showed efficacy in reducing depression scores at 3 weeks compared to 1 mg, but with notable adverse effects. The 10 mg dose did not significantly differ from the 1 mg dose. Further studies are needed to evaluate long-term efficacy and safety.
Carhart-Harris *et al*., 2021	United Kingdom	Double-blind, randomised controlled trial	*N* = 59 (29 male, 20 female) Mean age = 41 years Ethnicity = 52 (85%) participants were White	To explore the impact of therapeutic alliance and rapport on depression outcomes in psilocybin-assisted therapy	Two psilocybin dosing sessions	Quick Inventory of Depressive Symptomatology (QIDS), Scale To Assess the Therapeutic Relationship (STAR-P)	Psilocybin (25 mg)	Not specifically mentioned	Although the study authors failed to show a significant difference between the psilocybin and escitalopram groups at 6 weeks in the designated primary outcome measure, most secondary outcomes, including other depression severity scores, favoured the psilocybin group. Notably, the researchers highlighted that the acute subjective effects of psilocybin, such as emotional breakthroughs and mystical-type experiences, were strong predictors of positive clinical outcomes. These experiences were associated with more significant decreases in depression scores.
Murphy *et al*., 2022	United Kingdom	Phase 2, double-blind, randomised controlled trial	*N* = 29 (11 females) Mean Age = 42.8 years Ethnicity = 27 participants (93%) identified as White	Compare effects of psilocybin and escitalopram in treating depression	Two doses over 6 weeks	Quick Inventory of Depressive Symptomatology–Self-Report (QIDS-SR-16)	Psilocybin and Escitalopram	Similar incidence of adverse events in both groups	Larger and longer trials needed to compare psilocybin with established antidepressants
Davis *et al*., 2021	United States Of America	Randomised, waiting list–controlled clinical trial	*N* = 24 (16 women and 8 men) Mean age = 39.8 years Ethnicity = not assessed.	Investigate the effect of psilocybin therapy in patients with Major Depressive Disorder (MDD)	Two psilocybin sessions	Structured assessments (e.g., SCID-5, SCID-5 Screening Personality Questionnaire, SCID-5 Personality Disorders, and Personality Assessment Inventory).	Psilocybin (session 1: 20 mg/70 kg; session 2: 30 mg/70 kg)	Transient increase in blood pressure, challenging emotional experiences, mild to moderate transient headache	Psilocybin-assisted therapy is efficacious in producing large, rapid, and sustained antidepressant effects in patients with MDD
Madsen *et al*., 2020. *	Denmark.	The study involved PET imaging, psychological assessments, and a single oral dose of psilocybin.	*N* = 10 (4 females) Mean age = 28.4 Ethnicity = not assessed.	To evaluate the long-term effects of psilocybin on mindfulness and personality, and its relation to 5-HT2A receptor binding.	Single psilocybin session.	[11C]Cimbi-36 PET imaging was used.	Psilocybin (0.2–0.3 mg/kg).	Specific side effects were not detailed in the accessible parts of the document.	A single dose of psilocybin led to long-term increases in mindfulness and openness, without a consistent change in neocortical 5-HT2A receptor binding.
Carbonaro *et al*., 2018 *	United States Of America	A double-blind method was used.	*N* = 20 (gender not assessed) Mean age = 28.5 years. Ethnicity = 19 were Caucasian (95%) and one was Asian American.	The aim was to directly compare the effects of psilocybin and DXM in the same participants, focusing on alterations in subjective experience.	There were five experimental sessions, each lasting about 7 hours, and a final follow-up session.	Instruments included blood pressure cuffs, pupilometers, and various questionnaires like the Subjective Effects Questionnaire, Altered States of Consciousness (5D-ASC), States of Consciousness Questionnaire, Mysticism Scale, Psychological Insight Questionnaire, Challenging Experience Questionnaire, and Hallucinogen Rating Scale.	The drugs used were psilocybin (10, 20, and 30 mg/70 kg) and dextromethorphan HBr (400 mg/70 kg).	Notable side effects included nausea and emesis, particularly with DXM. No participant vomited after receiving placebo or lower doses of psilocybin, but 55% did after receiving 400 mg/70 kg DXM	The study found that high doses of psilocybin and DXM produced similar overall perceived strengths of drug effects. However, the 30 mg/70 kg dose of psilocybin often produced significantly greater effects than DXM on various subscales and questionnaires assessing mystical experiences, psychological insights, and visual effects.
Barrett *et al*., 2018 *	United States Of America	Double-blind, placebo-controlled, counterbalanced, crossover design	*N* = 20 (11 females) Mean Age = 28.5 years Ethnicity = 19 Caucasian, 1 Asian American	To compare cognitive effects of psilocybin and dextromethorphan (DXM)	5 sessions per participant, with varying doses of psilocybin and one high dose of DXM	Motor Praxis task;Memory;Working Memory and Vigilance;Executive Function and Overall Cognitive Impairment;The Penn Line Orientation Test (PLOT). Computerised Neurocognitive Battery (CNB);his involved the Digit Symbol Substitution Task, measuring executive function, mental flexibility, and associative learning, and the Mini-Mental Status Examination (MMSE).	Psilocybin (low, medium, high doses: 10, 20, 30 mg/70 kg) and DXM (high dose: 400 mg/70 kg)	Drug-induced impairment in cognitive domains; DXM showed greater cognitive impairment compared to psilocybin	Psilocybin and DXM differ in their cognitive effects. Psilocybin had greater effects on working memory, whereas DXM had more pronounced effects on episodic memory, response inhibition, and executive control. DXM use poses greater risks if abused

Note: The **Montgomery–Åsberg Depression Rating Scale (MADRS)** is a clinician-administered tool to assess depressive symptom severity. It focuses on core symptoms such as sadness, tension, reduced sleep, appetite, concentration, and inability to feel. Scores range from 0 to 6 per item, with higher scores indicating greater severity (Montgomery & Åsberg, 1979).

The **GRID-Hamilton Depression Rating Scale (GRID-HAMD)** is a standardised clinician-administered tool for assessing depression severity across mood, sleep, appetite, and physical symptoms. Higher scores reflect more severe depression, while lower scores suggest improvement (Williams, 1988; Williams et al., 2008).

The **Quick Inventory of Depressive Symptomatology–Self-Report (QIDS-SR-16)** evaluates depressive symptoms such as mood, sleep, weight, and energy through patient self-report. Scores indicate depression severity (Rush et al., 2003).

The **Beck Depression Inventory (BDI)** is a self-report tool assessing cognitive, emotional, and physical depression symptoms, with higher scores reflecting greater severity (Beck et al., 1961).

The **Sheehan Disability Scale (SDS)** measures functional impairment across work, social, and family domains, offering insights into how depressive symptoms interfere with daily life (Sheehan, 1983).

The **Five-Dimensional Altered States of Consciousness Rating Scale (5D-ASC)** assesses subjective altered states during psychedelic experiences. It evaluates dimensions such as oceanic boundlessness, visual restructuralization, and ego dissolution (Dittrich, 1998).

The **Positive and Negative Affect Schedule (PANAS)** measures mood states by assessing positive and negative affect dimensions, offering insights into emotional responses during interventions (Watson et al., 1988).

The **Generalised Anxiety Disorder Scale (GAD-7)** is a widely used self-report tool that evaluates the severity of generalised anxiety symptoms, with higher scores indicating greater severity (Spitzer et al., 2006).

The **Work and Social Adjustment Scale (WSAS)** measures functional impairment in work, social, and personal domains, providing an understanding of the impact of mental health conditions on daily life (Mundt et al., 2002).

The **EQ-5D-3L** is a standardised instrument for measuring health-related quality of life. It assesses mobility, self-care, usual activities, pain/discomfort, and anxiety/depression (EuroQol Group, 1990).

The **Digit Symbol Substitution Test (DSST)** measures cognitive functioning, including attention, processing speed, and executive function, often used in clinical studies involving neurological or psychological conditions (Wechsler, 1955).

The **Adjective Mood Rating Scale (AMRS)** evaluates mood changes, including calmness, energy levels, and happiness, and is often used in studies involving psychoactive substances (Janke & Debus, 1978).

The **Mystical Experience Questionnaire (MEQ)** measures mystical or spiritual experiences during interventions like psilocybin. It focuses on feelings of unity, transcendence, and sacredness (Barrett et al., 2015).

The **Emotional Breakthrough Inventory (EBI)** assesses emotional release and transformation, capturing moments of resolving struggles and gaining new perspectives during therapy (Roseman et al., 2019).

The **Big Five Inventory (BFI)** and the **Big Five Aspects Scale** assess personality traits across five dimensions: openness, conscientiousness, extraversion, agreeableness, and neuroticism (John & Srivastava, 1999).

The **Modified-Tellegen Absorption Scale** measures individuals’ openness to immersive and transformative experiences, often in psychedelic contexts (Tellegen & Atkinson, 1974).

The **Barratt Impulsivity Scale-Brief** evaluates impulsivity across cognitive, motor, and non-planning domains, commonly used in psychological studies (Patton et al., 1995).

The **Ruminative Response Scale (RRS)** measures the extent to which individuals focus on negative thoughts and emotions, often used in depression studies (Nolen-Hoeksema, 1991).

The **White Bear Suppression Inventory (WBSI)** assesses thought suppression tendencies, which are associated with mental health outcomes, including depression and anxiety (Wegner & Zanakos, 1994).

The **Visual Analogue Scale (VAS)** is a simple tool for measuring subjective experiences, such as mood or pain intensity, by having participants mark a point on a line representing their experience (Huskisson, 1974).

The **QLES-Q-SF** measures the degree of enjoyment and satisfaction individuals experience across various areas of daily life, including physical health, mood, work, and social relationships. It is widely used in psychiatric research and clinical trials to assess quality of life, particularly in depression and related disorders. (**Endicott, et al., 1993**).

### Geographical and demographic distribution

The majority of studies were conducted in North America (*n* = 12) and Europe (*n* = 10), with three studies involving participants from both regions. Across all studies, the combined sample included 852 participants, with a mean age of 39.1 years (range: 28.5 – 43.3). Due to inconsistent reporting, a standard error of the mean (SEM) could not be uniformly calculated.

### Methodological insights

Randomisation methods varied across studies and included computer-generated randomisation, block randomisation, and stratified randomisation. Blinding procedures, essential for minimising bias, were inconsistently reported. All studies were rated as 3–5 points (high-quality studies with low risk of bias) on the Jadad scale (Table 2). In Table 2, we evaluated three variables related to bias level: randomisation, blinding, and withdrawals/dropouts. Additionally, we indicated whether there was a reduction in depression.

**Table 2.** Methodological quality and risk of bias (Jadad Scale).

Author (year)	Randomisation		Blinding		Withdrawals and dropouts		
	Was the study described as randomised?	Was the method of randomisation appropriate?	Was the study described as blinding? a	Was the method of blinding appropriate?	Was there a description of withdrawals and dropouts?	**Total Score**	Decrease Depression
Aaronson *et al.,* 2025	**1**	**1**	**1**	**1**	**1**	**5**	Following a single 25 mg dose of psilocybin with psychological support, participants in the open-label trial showed significant reductions in depression. At week 1, 75% of patients met both response and remission criteria. By week 3, the study’s primary endpoint, 66.7% of participants met response criteria, and 41.7% met remission criteria. Although there was a drop at week 9 – with only 33% meeting response criteria – all of those responders were also in remission. At the study endpoint (week 12), 58.3% of participants met response criteria, and 25% remained in remission, indicating sustained effects. Self-rated depressive symptoms, measured using the QIDS-SR-16, were significantly reduced at weeks 1, 3, and 12, with large effect sizes (Cohen’s d ranging from 1.1 to 1.4).
Erritzoe *et al.,* 2024	**1**	**1**	**1**	**1**	**1**	**5**	Overall, while Psilocybin therapy (PT) and escitalopram treatment (ET) interventions were both associated with improvements in depressive symptom severity at 6-month follow-up, we observed greater benefits for psychological connectedness, general functioning, and existential meaning among PT patients. These results are considered preliminary due to missing data and limitations on visibility into treatment-seeking behaviour within the follow-up period.
Husain *et al*., 2023	**1**	**1**	**1**	**1**	**1**	**5**	It doesn’t explicitly state whether they measure decreased depression.
Skosnik *et al*., 2023	1	1	1	1	1	**5**	They found that increased auditory evoked theta power two weeks after a single dose of psilocybin, which correlated with improvements in depression measures, specifically the GRID-HAMD-17. This suggests that psilocybin may promote neuroplasticity and alleviate depressive symptoms.
Goodwin *et al*., 2023	1	1	1	1	0.5	**4.5**	Rapid Acting: Treatment differences were noticeable as early as Day 2 post-administration, indicating the rapid-acting nature of psilocybin. Clinician-Rated Improvement: The findings on patient-reported depression severity align with the results from the clinician-rated Montgomery-Åsberg Depression Rating Scale (MADRS), the primary efficacy measure in the study. The study also highlighted additional benefits, including reduced anxiety and increased positive affect, further supporting psilocybin’s potential in treating depression.
Ley *et al*., 2023	1	1	1	1	1	**5**	Not providing detailed results.
Weiss *et al*., 2023	1	0	1	1	1	**4**	The study reports a decrease in depression in both the psilocybin therapy (PT) and escitalopram treatment (ET) groups, based on changes in personality traits closely linked to depression and self-reported measures of symptom severity. Neuroticism, a key trait associated with depression, decreased significantly in both groups, with a greater reduction in the psilocybin group: □ = − 0.63 / B= −0.63 at week 6 and □ = − 0.47 / B= −0.47 at month 6 for PT, and □ = − 0.38 / B = −0.38 at week 6 and □ = − 0.46 / B= −0.46 at month 6 for ET. Introversion, linked to anhedonia and amotivation, decreased significantly in the psilocybin group (B = −0.38 B= −0.38 at week 6) but not significantly in the escitalopram group. Additionally, significant improvements in openness and absorption, traits linked to emotional flexibility and reduced depressive symptoms, were observed primarily in the psilocybin group. Despite these changes, no significant difference was found between the two conditions on the primary clinical outcome of depression severity, suggesting comparable efficacy of psilocybin therapy and escitalopram in reducing depression.
Goodwin *et al*., 2023	1	1	1	1	1	**5**	Showed a greater improvement from baseline in the MADRS total score 3 weeks post-psilocybin administration.
Raison, *et al*., 2023.	1	1	1	1	1	**5**	The mean score at baseline was 35.5 in the psilocybin group and 35.0 in the control group. At day 43, the psilocybin group experienced a greater reduction in the MADRS score than the control group, with a mean change difference of −12.3 points. The psilocybin group showed a 19.1-point reduction in the MADRS score from baseline to day 43, which is considered a substantial clinical improvement.
Sloshower *et al*., 2023 *	–	–	–	–	–	**-**	IDS-SR-16 showed a significant overall time effect, with a rapid decrease in depression scores one day after psilocybin administration that remained statistically significant through week 12.
Von Rotza *et al*., 2023	1	1	1	1	1	**5**	They observed that a single, moderate dose of psilocybin (0.215 mg/kg) led to significant reductions in depressive symptoms, measured using the Hamilton Depression Rating Scale (HDRS). Here are some key points from their findings: Significant improvement: Participants in the psilocybin group experienced a statistically significant improvement in depression scores compared to those in the placebo group. Effect size: The effect size was large (Cohen’s *d* = 1.0), indicating a substantial difference between the groups. Rapid onset: Similar to Goodwin *et al*. (2023); Von Rotza *et al*. (2023) observed that the antidepressant effects of psilocybin appeared relatively quickly.
Holze *et al*., 2022	1	1	1	0.5	1	**4.5**	This study just checked healthy participants.
Barba *et al*., 2022	1	1	1	1	1	**5**	Participants with major depressive disorder were randomly assigned to receive either psilocybin or escitalopram. The researchers found that, compared to those receiving escitalopram, participants in the psilocybin group experienced significantly greater reductions in both rumination and thought suppression.
Gukasyan *et al*., 2022 **	–	–	–	–	–	**-**	GRID-HAMD score showed a decrease from a mean of 22.8 at baseline to 8.7 at week 1 and remained low through the 12-month follow-up with a Cohen d of 2.4 at 12 months.
Daws *et al*., 2022.	1	0.5	1	1	1	**4.5**	Reported that after treatment, BDI scores were significantly reduced at week 1 (mean difference = −21 points, Cohen’s *d* = 1.78) and remained reduced at 6 months (mean difference = −14.19 points, Cohen’s *d* = 1.07).
Goodwin *et al*., 2022	1	1	1	1	1	**5**	The mean change in MADRS score from baseline to week 3 was −12.0 for the 25 mg psilocybin group, −7.9 for the 10 mg group, and −5.4 for the control group. The difference between the 25 mg group and the control group was statistically significant (*p* < 0.001).
Murphy *et al*., 2022	1	1	1	1	0	**4**	They found that a stronger therapeutic alliance before the first psilocybin session predicted greater reductions in depression scores (*β* = 0.48, *p* < 0.001). They also found that a weaker alliance ahead of the second session predicted higher depression scores at the study’s endpoint (*β* = −0.49, *p* < 0.001). The study used data from a double-blind, randomised controlled trial (DB-RCT) where individuals with MDD received two treatment sessions of psilocybin (25 mg) along with psychological support.
Carhart-Harris *et al*., 2021	1	1	1	1	1	**5**	No significant difference between groups at week 6. Patients who received psilocybin experienced significant reductions in depressive symptoms as measured by the 16-item Quick Inventory of Depressive Symptomatology (QIDS-SR-16) at the 6-week endpoint. More participants in the psilocybin group were classified as responders (21 out of 30) compared with the escitalopram group (14 out of 29). A responder was defined as a patient who had a clinically significant reduction in their rumination score at the 6-week follow-up.
Davis *et al*., 2021	1	0.5	1	0	1	**3.5**	Higher scores indicate greater severity of depression, found the mean score decreased from 22.8 at baseline to 8.7 at week 1 and 8.9 at week 4 post-session - Cohen *d* = 2.3.
Madsen *et al*., 2020.***	–	–	–	–	–	**-**	It does not specifically mention depression scores or scales.
Carbonaro *et al*., 2018	1	1	1	0.5	1	**4.5**	Does not report on depression levels, as its primary focus was on subjective effects, not therapeutic outcomes.
Barrett *et al*., 2018	1	1	1	0.5	0.5	**4**	The study does not explicitly report direct measurements or reductions in depression symptoms. It primarily focuses on neurocognitive performance and subjective drug experiences in a double-blind comparison with dextromethorphan (DXM).

* This exploratory, placebo-controlled, fixed-order trial investigated the effects of psilocybin-assisted therapy for major depressive disorder. While the trial was placebo-controlled, it utilised a fixed-order design, meaning that all participants received both psilocybin and placebo in a predetermined sequence, potentially introducing order effects.

** Not specified like a RTCs.

*** Do not explicitly state the study’s design. They lack crucial information about the methodology, including whether there was a control group or randomisation.

**Table 3.** PICO framework

PICO element	Inclusion Criteria	Exclusion criteria
Participants or population	Adults with depression, MDD, or TRD	Under 18 years or any diagnosis other than depression.
Intervention or exposure	Psilocybin treatment or antidepressants	–
Comparators or Control (where relevant)	Healthy participants or control conditions will include patients without depression who receive a placebo in the clinical process and comparison with other drugs, such as Ketamine, Niacin, or Mirtazapine.	–
Outcomes	Measures of emotional state (i.e., biological and subjective). Measures of neural activity (i.e., results from EEG, fMRI, or other methods). Measures of subjective interpretation (i.e., preference and familiarity).	–
Study design	RTC’s	Systematic reviews, meta-analyses, case reports, animal studies, editorials, expert opinions, conference abstracts

Abbreviations:.

**MDD**: Major Depressive Disorder; **PICO**-P- Participants, I- Intervention, C- Comparators or control, O-Outcomes; **TRD**:Treatment Resistant Depression.

**EEG** – Electroencephalography; **fMRI** – Functional Magnetic Resonance Imaging..

### Depression measurement tools and quantitative insights

**Figure 2.**
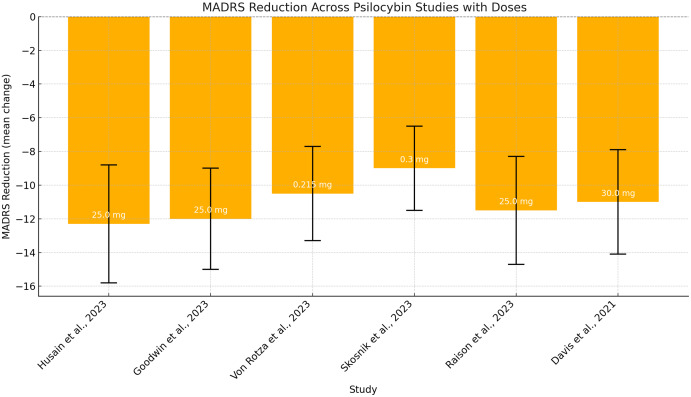
MADRS score changes following psilocybin administration at different doses (mg/kg) across diferent studies.

The Montgomery–Åsberg Depression Rating Scale (MADRS) was the most commonly employed tool for evaluating psilocybin’s efficacy, as reported in studies by Goodwin *et al*., (2022, 2023), Raison *et al*. (2023); Skosnik *et al*. (2023); Von Rotza *et al*. (2023); Weiss *et al*. (2023); and Husain *et al*. (2023) (See Fig. 2). Other depression scales used included the GRID-Hamilton Depression Rating Scale (GRID-HAMD) in studies by Davis *et al*. (2021) and Gukasyan *et al*. (2022) and the Beck Depression Inventory (BDI) in studies by Gukasyan *et al*. (2022) and von Rotza *et al*. (2023).

Additional measures, such as the Mystical Experience Questionnaire (MEQ) and Emotional Breakthrough Inventory (EBI), provided insights into psilocybin’s psychological mechanisms, highlighting emotional release and transformative experiences as influencing outcomes.

### Efficacy of psilocybin in treating depression

#### Major depressive disorder (MDD)

Two RCTs demonstrated that psilocybin-assisted therapy produced substantial and lasting antidepressant effects in MDD, with large effect sizes observed when comparing high vs. low doses (Davis *et al*., 2021; Gukasyan *et al*., 2022). Studies also showed significant reductions in GRID-HAMD scores over time (Barrett *et al*., 2015; Carbonaro *et al*., 2020; Davis *et al*., 2021).

A trial comparing psilocybin to an active placebo (niacin) reported significantly greater MADRS score reductions in the psilocybin group (−12.3 to day 43; −12.0 to day 8), along with improved functional impairment, though remission rates were comparable (Raison *et al*., 2023).

A smaller RCT using a 0.215 mg/kg dose reported 58% of participants responding on MADRS and 54% on BDI by day 14, compared to 16% and 12% in the placebo group (von Rotza *et al*., 2023). However, another RCT comparing psilocybin to escitalopram showed no significant difference in QIDS-SR-16 scores at week 6, although secondary outcomes favoured psilocybin (Carhart-Harris *et al*., 2021).

#### Treatment-resistant depression (TRD)

One RCT reported a −14.9 point MADRS reduction in TRD patients already on SRIs at week 3, with 42.1% of participants achieving both response and remission. Effects were observed as early as day 2 and sustained over time (Goodwin *et al*., 2022; Goodwin *et al*., 2023).

Another study found that a 25-mg dose produced the most significant effect (−12.0 at week 3), compared to −5.4 in controls. The 10-mg dose showed a moderate effect (−7.9), which was not statistically significant (Goodwin *et al*., 2022).

A large trial involving 233 TRD patients further supported these findings: the 25-mg group showed statistically significant improvement across measures of depression, anxiety, affect, functioning, and quality of life (Carhart-Harris *et al*., 2016, 2021; Goodwin *et al*., 2022; Davis *et al*., 2021; Grob *et al*., 2011; Griffiths *et al*., 2016; Ross *et al*., 2016).

#### Role of psychotherapy and set/Setting

Multiple studies (Davis *et al*., 2021; Goodwin *et al*., 2022; Raison *et al*., 2023; von Rotza *et al*., 2023; Aaronson *et al*., 2025) emphasise that preparatory and integration therapy enhance both the safety and efficacy of psilocybin treatment. These components typically include structured preparation and post-session integration, which help participants process their experiences and mitigate adverse effects (Swieczkowski *et al*., 2025).

Griffiths *et al*. (2016) and Carhart-Harris *et al*. (2021) noted that psychological support appears especially important for managing high-dose effects. However, participants receiving active placebos (e.g., low-dose psilocybin or niacin) may still experience physiological sensations that amplify expectancy effects, complicating blinding and possibly inflating placebo responses (Aday *et al*., 2022; Barstowe, A. and Kajonius, P.J. 2024 ‘Masking influences: a systematic review of placebo control and masking in psychedelic studies’, Journal of Psychoactive Drugs, published online 6 November, pp. 1–11. https://doi.org/10.1080/02791072.2024.2424272 &, ).

Although evidence supports these psychotherapeutic elements, the optimal format, timing, and intensity remain undefined. This highlights the need for standardised interventions within the “set and setting” framework.

#### Dose–Response variability across diagnoses

Effective psilocybin dosing across patient groups variably complicates interpretation. Meta-analyses (Li *et al*., 2022; Perez *et al*., 2023) suggest lower doses may alleviate depressive symptoms in secondary depression, especially with comorbid anxiety. In contrast, TRD patients often require higher doses, likely due to pharmacoresistance and neurobiological factors. This dose heterogeneity emphasises the need for individualised treatment protocols and further studies that clarify dose–response relationships by diagnosis.

#### Involvement of the 5HT_2_A receptor in psilocybin’s antidepressant effects

The 5-hydroxytryptamine 2A (5-HT_2_A) receptor is widely recognised as the principal pharmacological target of psilocybin via its active metabolite, psilocin. Psilocin acts as a partial agonist at 5-HT_2_A receptors, which are abundantly expressed in cortical regions involved in mood regulation, including the prefrontal and posterior cingulate cortices (Nichols, 2016; Ray, 2010). Activation of this receptor is considered essential for both the acute psychedelic effects and the therapeutic outcomes observed with psilocybin administration (Vollenweider & Kometer, 2010).

Direct evidence of 5-HT_2_A receptor involvement comes from human PET imaging studies. Madsen *et al*. (2019) demonstrated that oral psilocybin leads to up to 72% 5-HT_2_A receptor occupancy, with occupancy levels closely correlating with plasma psilocin concentrations and the subjective intensity of the psychedelic experience. Similarly, Vollenweider *et al*. (1998) showed that pretreatment with ketanserin, a selective 5-HT_2_A antagonist, blocks the subjective and neurophysiological effects of psilocybin, reinforcing the receptor’s central role.

Although much of the evidence for neuroplasticity-related pathways such as TrkB and mTOR comes from broader psychedelic research, Vargas *et al.,* (2023) demonstrated that psilocin, but not serotonin, can activate intracellular 5-HT_2_A receptors, particularly those located in the Golgi apparatus, leading to structural plasticity. These intracellular receptor populations may play a distinct role in the cellular mechanisms underlying psilocybin’s therapeutic potential.

These findings support the conclusion that activation of 5-HT_2_A receptors, both membrane-bound and possibly intracellular, is a key mediator of psilocybin’s antidepressant effects. While direct receptor binding was not assessed in most clinical trials reviewed here, the observed clinical efficacy and sustained psychological changes are consistent with known 5-HT_2_A receptor pharmacology. This aligns with a growing body of evidence suggesting that psilocybin’s therapeutic action arises primarily from 5-HT_2_A receptor activation and the downstream modulation of neural circuits involved in affect regulation.

## Discussion

The therapeutic potential of psilocybin in treating depression has been extensively studied, with more than 10 investigations evaluating its effects on depressive symptoms at multiple time points post-administration. Clinical trials consistently demonstrate that psilocybin induces rapid and significant reductions in depressive symptoms, often following just one or two treatment sessions. For instance, Carhart-Harris *et al*. (2021) compared psilocybin with escitalopram using the QIDS-SR-16 scale and found that while no statistically significant differences were observed between groups at six weeks, secondary outcomes favoured psilocybin. Notably, 57% of psilocybin-treated patients achieved remission compared to 28% in the escitalopram group. Similarly, Davis *et al*. (2021) reported substantial reductions in GRID-HAMD scores, with a mean score decrease from 22.9 at baseline to 8.0 at one week post-treatment, corresponding to a large effect size (Cohen’s *d* = 2.5).

Psilocybin, a serotonergic compound found in certain mushrooms, has reemerged in psychiatric research over the past decade. From a pharmacokinetic standpoint, psilocybin acts as a prodrug, undergoing rapid dephosphorylation in the gut and liver to produce psilocin, the active metabolite responsible for its central nervous system effects (Hasler *et al*., 2004; Meshkat *et al*., 2025). Psilocin crosses the blood–brain barrier efficiently, reaching peak plasma concentrations within 1.5 to 2 hours after oral ingestion, with a half-life ranging from approximately 1.8 to 4.8 hours depending on dose and study population (Madsen *et al*., 2019; Meshkat *et al*., 2025). Psilocin primarily acts as a partial agonist at the 5-HT_2_A receptor, but also engages 5-HT_1_A and 5-HT_2_C receptors, contributing to its complex psychoactive and therapeutic profile (Ray, 2010; Nichols, 2016).

Importantly, a distinction must be made between the acute and long-term effects of psilocybin. Acutely, users experience altered perception, time distortion, and emotional shifts – effects that are temporally linked to psilocin’s peak brain activity (Carhart-Harris *et al*., 2012). However, Griffiths *et al*. (2016) and Davis *et al*. (2021) report that a single or few doses can lead to long-term improvements in mood, anxiety, and existential distress, persisting for weeks to months. These enduring effects are hypothesised to result from downstream neurobiological mechanisms, including enhanced emotional plasticity, altered default mode network activity, and increased synaptogenesis (Ly *et al*., 2018; Carhart-Harris & Friston, 2019).

Pharmacodynamically, psilocin’s agonism at the 5-HT_2_A receptor has been linked to increased cortical glutamate release and enhanced synaptic plasticity, which may underlie the rapid and durable antidepressant effects observed in clinical settings (Vollenweider & Preller, 2020). Recent research has emphasised the relevance of biased agonism, whereby ligands selectively activate specific intracellular signalling pathways at the same receptor. Evidence suggests that psilocin and other psychedelics may preferentially activate β-arrestin-mediated pathways over G protein-dependent signalling at the 5-HT_2_A receptor, potentially contributing to their unique therapeutic profile (Wacker *et al*., 2017; Wallach *et al*., 2023). This functional selectivity has prompted interest in developing non-hallucinogenic 5-HT_2_A modulators, such as lisuride or newer analogues, that may retain antidepressant properties without eliciting psychedelic effects (Nichols, 2016; Wulff *et al.,* 2023).

**Figure 3.**
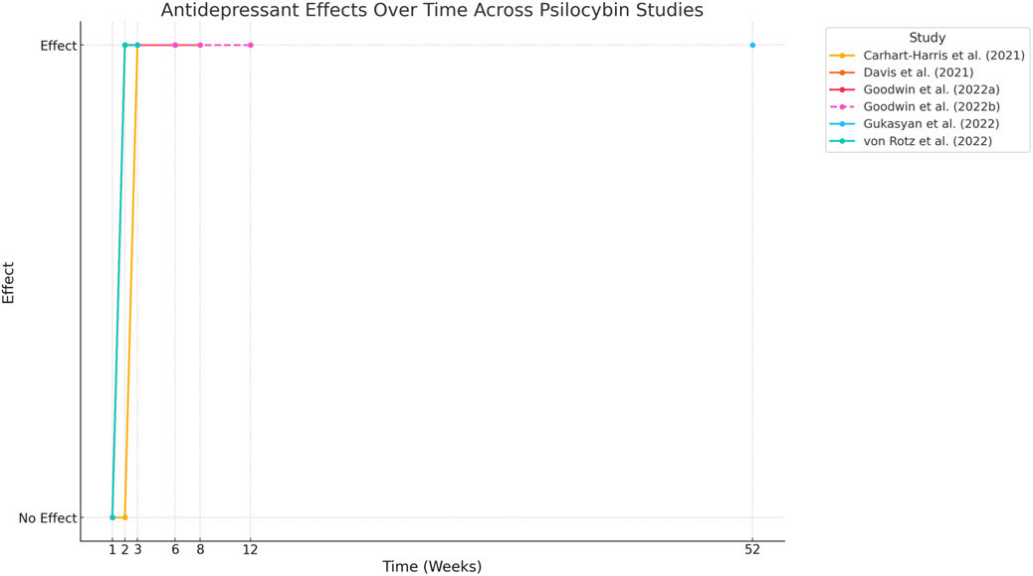
Time Course of psilocybin antidepressant (follow-up up to 52 weeks).

Research also highlights the durability of psilocybin’s effects. Goodwin *et al*., (2022, 2023) showed significant reductions in MADRS and QIDS-SR-16 scores as early as day two post-treatment, with these effects sustained through weeks three and twelve. Gukasyan *et al*. (2022) extended these findings, reporting large effect sizes (Cohen’s *d* = 2.4) and high response and remission rates up to 12 months (52 weeks) post-treatment (see Fig. 3).

Furthermore, Raison *et al*. (2023) and von Rotz *et al.*, (2022) observed rapid reductions in MADRS scores within 8–14 days and significant improvements compared to placebo.

Beyond depression, psilocybin therapy has shown broader mental health benefits, including reductions in anxiety (Grob *et al*., 2011; Ross *et al*., 2016), improved mindfulness (Madsen *et al*., 2020), and changes in personality traits such as increased Openness and decreased Neuroticism (Weiss *et al.,* 2025). These psychological outcomes may play a critical role in sustaining therapeutic gains.

Another topic of interest is the interaction between psilocybin and serotonin reuptake inhibitors (SRIs). Barbut-Siva et al. (2024) found no significant differences in subjective psychedelic experiences or therapeutic outcomes between individuals on SRIs and those without, suggesting that concurrent SRI use does not negate psilocybin’s benefits. These findings support the potential feasibility of inclusive treatment protocols.

Methodologically, studies have adopted diverse designs and objectives. Husain *et al*. (2023) examined the combination of psilocybin and risperidone for treatment-resistant depression. Skosnik *et al*. (2023) investigated psilocybin’s effects on neuroplasticity via EEG, while Goodwin *et al*. (2023) assessed dosing efficacy. Ley *et al*. (2023) compared subjective effects across several psychedelics under controlled settings.

Finally, the necessity of the psychedelic experience itself for therapeutic benefit remains debated. Studies report a positive correlation between mystical-type experiences and clinical outcomes (Roseman *et al*., 2018; Romeo *et al*., 2025; Menon *et al.,* 2025 , raising questions about whether these subjective effects are essential or incidental. This has led to increasing exploration of biased signalling mechanisms, as described above, and the development of novel compounds that may uncouple therapeutic effects from hallucinogenic states (Urban *et al*., 2007; Cameron *et al*., 2021).

Due to the considerable heterogeneity in study designs, populations, interventions, and outcome measures, a meta-analytic approach was not feasible. Instead, this narrative synthesis aimed to capture the variability and evolving trends in psilocybin research. This heterogeneity reflects both the emerging nature of this field and the diverse methodologies employed across trials.

Overall, these findings contribute to a growing evidence base supporting psilocybin’s therapeutic utility. By integrating pharmacokinetic insights, neurobiological mechanisms, and clinical outcomes, this review emphasises the importance of optimising treatment protocols and understanding individual variability. Future directions include identifying optimal dosing strategies, clarifying the role of subjective experiences, and advancing the development of non-hallucinogenic analogues, each of which may further establish psilocybin as a transformative agent in psychiatric care. Importantly, these insights converge on the central role of 5-HT_2_A receptor activation as a key mechanism underlying psilocybin’s antidepressant effects, reinforcing its relevance as both a therapeutic target and a focal point for future research.

### Safety concern

While the therapeutic benefits of psilocybin are increasingly supported by clinical trials, its safety profile warrants careful scrutiny. Although most studies report only mild and transient adverse effects, such as nausea, anxiety, or headaches, there is emerging evidence of more serious risks in some individuals. Notably, Goodwin *et al*. (2022) documented transient increases in suicidal ideation in a subset of participants, underscoring the need for comprehensive psychiatric screening and continuous monitoring. Moreover, the intensity and emotional depth of the psychedelic experience may pose psychological risks if administered outside of a controlled and supportive setting. Therefore, safe administration requires more than appropriate dosing; it depends critically on trained psychological support, preparation, and integration practices that reduce the likelihood of adverse reactions and enhance therapeutic outcomes.

### Challenges and strategies in dose standardisation

Standardising psilocybin dosing presents a complex challenge due to significant interindividual variability in pharmacokinetics. Psilocybin is rapidly converted into psilocin, whose absorption and metabolism are influenced by cytochrome P450 enzymes, particularly CYP2D6 and CYP3A4 (Thomann *et al*., 2024; Meshkat *et al*., 2025). Peak plasma concentrations occur between 1.5 and 4 hours post-ingestion, with elimination half-lives extending up to 4.8 hours depending on the dose and individual metabolic profiles (Madsen *et al*., 2019). This variability implies that fixed-dose regimens, such as the commonly used 25 mg oral dose, may yield uneven effects across individuals. Pharmacokinetic modelling and identifying predictive biomarkers may help tailor dosing more precisely, optimising safety and efficacy.

### Toward safer and more inclusive dosing protocol

Establishing robust and inclusive treatment protocols also involves addressing the concurrent use of antidepressants. Recent findings suggest that selective serotonin reuptake inhibitors (SRIs) do not significantly diminish the subjective or therapeutic effects of psilocybin (Barbut-Siva *et al.,* 2024), supporting the feasibility of inclusive approaches that do not require antidepressant discontinuation. This is particularly important for patients with severe or unstable depression, where withdrawal from SRIs could pose additional risks. Furthermore, research into varied dosing paradigms, from single high-dose sessions to repeated moderate dosing, offers promising avenues for individualised care (Goodwin *et al*., 2023; Ley *et al*., 2023). These emerging strategies, along with ongoing efforts to develop non-hallucinogenic analogues, mark critical steps toward integrating psilocybin into psychiatric practice with greater safety, flexibility, and therapeutic reach.

## Conclusion

The data collected demonstrated that there is evidence that indicates promising therapeutic potentials of psilocybin for depression. It also underscores the necessity for further studies. Additional research is needed to fully understand psilocybin’s long-term effects, optimal dosing, safety in diverse populations, and mechanisms of action, especially regarding its interaction with the 5-HT2A receptors. This will enable a more comprehensive assessment of its efficacy and integration into clinical practice for treating depression and other mental health disorders. This body of research marks a significant step towards understanding and potentially revolutionising the treatment of depression, especially in cases where traditional therapies have been ineffective.

### Limitations of this study

The study has limitations, as the included studies have small sample sizes, short follow-up periods, and a lack of diversity among the studied populations. These factors restrict the generalisability of the findings across different ethnic, cultural, and age groups. Expanding participant recruitment to include a broader range of populations is crucial for understanding potential variations in the efficacy and safety of psilocybin.

While some studies report sustained effects of psilocybin treatment lasting up to 12 months, most only extend follow-up beyond a few weeks or months. The durability of psilocybin’s antidepressant effects and the potential for relapse have not been fully explored. Future research should prioritise long-term follow-up to assess outcomes and safety over several years.

There is also considerable variability in dosing regimens, session frequency, and adjunctive therapies across studies, complicating direct comparisons. Standardising protocols, including dose ranges and session structures, is necessary to establish consistent findings and optimise clinical applications.

More extensive, diverse, and rigorously designed trials must confirm and expand upon existing findings. The scope of future research should involve a variety of methodologies, a wider range of populations, and long-term outcome assessments to thoroughly evaluate the efficacy and safety of psilocybin in treating depression.

## Future directions

Exploring the individual differences in response to psilocybin, focusing on the influence of personality traits, 5-HT2AR binding, and other relevant factors. Studies (Carhart-Harris *et al*., 2016; Barrett *et al*., 2017b; MacLean *et al*., 2011; Griffiths *et al*., 2018; Madsen *et al*., 2020) indicate variability in how individuals respond to psilocybin. Future research should investigate the role of personality traits, such as openness, and changes in 5-HT2AR levels as potential moderators of treatment effects. Additionally, it is essential to examine factors like symptom severity, depression subtypes, comorbidities, and prior 5-HT2AR stimulation.

## Supporting information

Andrade et al. supplementary material Andrade et al. supplementary material

## Supplementary material

The supplementary material for this article can be found at https://doi.org/10.1017/neu.2025.10039.
