# Supplementary material for: Therapeutic Use of Psilocybin in Depression: a Systematic Review of Clinical Evidence
Source: Acta Neuropsychiatr. 2025 Sep 3;37:e86. doi: 10.1017/neu.2025.10039 (PMC13130258; doi:10.1017/neu.2025.10039)
Supplement: Andrade et al. supplementary material [file S0924270825100392sup001.zip › Table 1 - June 2025.docx]

| **Author (year)** | **Country** | **Methods** | **Sample** | **Aims** | **Number of Sessions** | **Instruments** | **Type of drug** | **Related side effects** | **Conclusion** |
| --- | --- | --- | --- | --- | --- | --- | --- | --- | --- |
| Aaronson et al., 2024 | United States | 12-week, open-label clinical trial involving a single 25 mg dose of synthetic psilocybin with preparatory and integration psychotherapy. | N = 12 (6 male, 6 female);  Mean age = 40.6 years  Ethnicity = 100% White. | To assess the safety and antidepressant efficacy of a single dose of psilocybin in individuals with severe treatment-resistant depression (TRD) | 3 preparatory therapy sessions;  1 psilocybin dosing session (8–9 hours);  3 integration sessions post-dosing;  Regular follow-ups at weeks 1, 2, 3, 6, 9, and 12. | Montgomery-Åsberg Depression Rating Scale (MADRS); Quick Inventory of Depressive Symptoms – Self-Rated (QIDS-SR-16); General Anxiety Disorder-7 (GAD-7); Quality of Life Enjoyment and Satisfaction Questionnaire (QLES-Q-SF); Work and Social Adjustment Scale (WSAS); 5D-Altered States of Consciousness Scale (5D-ASC); Challenging Experience Questionnaire (CEQ); Mystical Experience Questionnaire (MEQ). | Synthetic psilocybin (25 mg, COMP360 formulation by COMPASS Pathways) | Mild headache (16.7%);  Moderate insomnia (2 patients);  Moderate worsening of depression in 1 patient;  Psychomotor agitation (1 case);  No serious adverse events reported. | Supports the safety and potential efficacy of psilocybin for severe TRD, with significant symptom improvement observed in most participants. |
| Erritzoe et al,. 2024 | United Kingdom | 6-month follow-up of a phase 2, double-blind, randomized controlled trial | N = 59 (29 male, 20 female)  Mean age = 41 years  Ethnicity = 52 (85%) participants were White. | To compare the long-term (6 months) effects of psilocybin therapy versus escitalopram on depression severity, social functioning, psychological connectedness, and meaning in life, and to assess their sustainability after intensive treatment. | Psilocybin group: 2 doses administered approximately 3 weeks apart, with around 20 hours of in-person psychological support across the treatment.  Escitalopram group: 6-week course of daily medication ("escitalopram" at 10 mg and 20 mg) with psychological support. | 16-item Quick Inventory of Depressive Symptomatology – Self-Report (QIDS-SR-16); Work and Social Adjustment Scale (WSAS); Watts’ Connectedness Scale (WCS);  Meaning in Life Questionnaire (MLQ);  Flourishing Scale (FS) | Psilocybin (1 mg)  Escitalopram (10 mg, then 20 mg) | Side effects not detailed | Both treatments resulted in sustained reductions in depression severity over six months. Psilocybin therapy was associated with greater improvements in social functioning, connectedness, and meaning in life, beyond symptom reduction. |
| Husain et al., 2023 | Canada | Double-Blind Proof-of-Concept Randomised Controlled Trial | N=60  Mean Age = not assessed. Ethnicity = not assessed. | To block the psychedelic effects of psilocybin and provide data on its antidepressant effect. | Not specify the number of days, but is 12 hours of psychotherapy in each session. | Montgomery–Åsberg Depression Rating Scale (MADRS), 5-Dimensional Altered States of Consciousness Rating Scale (5D-ASC), among others | Psilocybin (25 mg) and Risperidone (1 mg) | Side effects associated with the combination of psilocybin and risperidone are examined | The research may increase the acceptability and access to psilocybin for TRD without needing psychedelic experiences |
| Skosnik et al., 2023 | United States Of America | Double-blind, placebo-controlled within-subject study | N= 19  Mean Age = not assessed.  Ethnicity = not assessed. | Investigate the effects of psilocybin on EEG correlates of neuroplasticity and its relationship with depression symptoms. | Two (placebo followed by psilocybin) | Electroencephalography (EEG), GRID Hamilton Rating Scale for Depression-17 (GRID-HAM-D-17), and MADRS) | Psilocybin (0.3 mg/kg) and placebo | Not specifically mentioned | Psilocybin increased auditory evoked theta power two weeks post-administration, correlating with improvements in depression symptoms, indicating potential neuroplastic changes in the brain. |
| Goodwin et al., 2023 | 22 sites across 7 countries in Europe and North America | Phase 2, multicenter, international double-blind trial | N= 233 (52% females)  Mean age = 39.8 years  Ethnicity = 92% were White. | Evaluate the efficacy of single-dose psilocybin in treatment-resistant depression. | Single dose | Various, including QIDS-SR-16, PANAS, GAD-7, SDS, WSAS, EQ-5D-3L, DSST | Psilocybin (25 mg, 10 mg, and 1 mg doses) | Adverse events in 66-84% of participants across doses | Single-dose psilocybin showed benefits in reducing depression and anxiety symptoms, and in improving functioning and quality of life in treatment-resistant depression |
| Ley et al., 2023* | Switzerland | Double-blind placebo-controlled crossover design with four experimental test sessions | N= 32 (16 men and 16 women) Mean age = 29  Ethnicity = not assessed. | Compare the acute effects of mescaline, LSD, and psilocybin. | Four sessions (300 mg or 500 mg mescaline, 100 µg LSD, 20 mg psilocybin, and placebo) | Adjective Mood Rating Scale (AMRS), and the 5 Dimensions of Altered States of Consciousness (5D-ASC) scale. | Mescaline hydrochloride (99.3% purity), LSD base (>99% purity), and psilocybin | Mescaline, LSD, and psilocybin were found to be tolerable when used at psychoactive-equivalent doses | No evidence of qualitative differences in altered states of consciousness induced by equally strong doses of mescaline, LSD, and psilocybin. Differences in pharmacological profiles do not translate into relevant differences in the subjective experience. |
| Weiss et al., 2023 | United Kingdom | Phase 2 double-blind randomized active comparator-controlled trial | N=59 (29 male, 20 female)  Mean age = 41 years  Ethnicity = 52 (85%) participants were White. | To examine changes in personality in relation to PT and ET. | Core 6-week trial period | Big Five Inventory, Big Five Aspects Scale, Modified-Tellegen Absorption Scale, Barratt Impulsivity Scale-Brief | Psilocybin and Escitalopram | Side effects not detailed | Both PT and ET resulted in personality changes consistent with improved mental health. No significant between-group differences, except for a trend in absorption changes favoring PT. |
| Goodwin et al., 2023 | International (Ireland and the United States) | Phase II exploratory fixed-dose open-label study | N = 19  Mean Age = not assessed.  Ethnicity = not assessed. | To explore the safety, tolerability, and efficacy of synthetic psilocybin (COMP360) as an adjunct to selective serotonin reuptake inhibitors (SSRIs) in treatment-resistant depression (TRD) patients. | Single administration session, followed by three weeks of follow-up. | Montgomery-Åsberg Depression Rating Scale (MADRS), Clinical Global Impression–Severity (CGI-S), Generalized Anxiety Disorder 7-item (GAD-7). | Synthetic psilocybin (COMP360) alongside SSRIs. | Mostly mild, including headaches and transient blood pressure increase; no serious treatment-emergent adverse events reported. | Psilocybin, when used alongside SSRIs, showed a favorable safety profile and potential efficacy in reducing symptoms of TRD. Further large-scale trials are recommended to confirm these findings. |
| Raison, et al., 2023. | Conducted in the United States. | Randomized, placebo-controlled clinical trial. | N = 104 (52 women)  Mean age = 41.1 years  Ethnicity = not assessed. | To evaluate the antidepressant effects and safety of a single dose of psilocybin compared to niacin in patients with Major Depressive Disorder (MDD). | One dosing session with follow-ups. | Montgomery-Åsberg Depression Rating Scale (MADRS), Sheehan Disability Scale (SDS). | Synthetic psilocybin (25 mg) and niacin as a placebo. | Headaches, nausea, and visual perceptual effects. | Psilocybin treatment led to a statistically significant reduction in depressive symptoms compared to niacin, with rapid onset and sustained effects over six weeks. |
| Sloshower et al., 2023 | United States Of America | Placebo-controlled, within-subject, fixed-order study | N = 19  Mean age= not assessed.  Ethnicity = not assessed. | To assess the efficacy of psilocybin-assisted therapy in treating major depressive disorder. | 2 dosing sessions, 8 psychotherapy sessions. | GRID-Hamilton Depression Rating Scale, Quick Inventory of Depressive Symptomatology–Self-Report | Psilocybin (0.3 mg/kg) and placebo (microcrystalline cellulose) | Mild to moderate transient headache, anxiety, and dysphoria; no severe adverse events. | Psilocybin-assisted therapy showed potential benefits in treating major depression, with significant improvements in depression and anxiety scores and quality of life. |
| von Rotza et al., 2023 | Switzerland | Randomized, double-blind, placebo-controlled trial | N= 52  Mean age = not assessed.  Ethnicity = not assessed. | Assess the effectiveness of a single moderate dose of psilocybin in treating MDD | One session, with follow-up assessments | Montgomery-Åsberg Depression Rating Scale (MADRS), Beck Depression Inventory (BDI) | Psilocybin (0.215 mg/kg body weight) and placebo | Headaches, nausea, no severe adverse events | Psilocybin significantly reduced depressive symptoms compared to placebo, with effects lasting for at least two weeks. The treatment was well-tolerated with mild side effects. |
| Holze et al., 2022 * | Switzerland | Double-blind, randomized, placebo-controlled crossover design | N= 28 (14 men, 14 women). Mean age  = 35 years  Ethnicity = not assessed. | Compare acute subjective, autonomic, and endocrine effects of LSD and psilocybin | 5 sessions per participant | Visual Analog Scales (VASs)  Adjective Mood Rating Scale (AMRS)  5D-ASC scale  States of Consciousness Questionnaire  Blood pressure, heart rate, and body temperature measurements  Pupil size assessment  List of Complaints (LC)  Plasma concentrations (cortisol, prolactin, oxytocin, BDNF) | LSD (100 and 200 µg), Psilocybin (15 and 30 mg) | Increased heart rate, blood pressure, body temperature, pupil size, and adverse events | LSD and psilocybin produced comparable subjective and autonomic effects, with differences in duration. |
| Barba et al., 2022 | United Kingdom | Randomized clinical trial | N = 59 (29 male, 20 female)  Mean age = 41 years  Ethnicity = 52 (85%) participants were White. | Compare the effects of psilocybin and escitalopram on rumination and thought suppression in major depressive disorder | Psilocybin: 2 sessions; Escitalopram: 6 weeks of treatment | Ruminative Response Scale (RRS), White Bear Suppression Inventory (WBSI), Quick Inventory of Depressive Symptomatology Self-Report (QIDS-SR-16) | Psilocybin, Escitalopram | The overall incidence of adverse events was similar between the psilocybin and escitalopram groups, and no serious adverse events occurred. It is noted that the side-effect profile of psilocybin was less diverse than that of escitalopram and was superior in certain domains, including anxiety, dry mouth, sexual dysfunction, and emotional function. | Psilocybin showed greater impact on reducing rumination and thought suppression compared to escitalopram in the treatment of depression. |
| Gukasyan et al., 2022 | United States Of America | Randomized waiting-list controlled study | N = 24 (67% were female)  Mean age = 39.8 years  Ethnicity= 22(92%) were Caucasian, one identified as Black and another as Asian. | To examine the efficacy and safety of psilocybin through 12 months in participants with moderate to severe MDD who received psilocybin. | Two doses of psilocybin. | GRID-Hamilton Depression Rating Scale (GRID-HAMD), Quick Inventory of Depressive Symptoms (QIDS), Beck Depression Inventory II (BDI-II) | Psilocybin | No serious adverse events, low suicidal ideation, no instances of self-injurious behavior, no reported use of psilocybin or other psychedelics outside of the study, no participant met criteria for HPPD. | Psilocybin-assisted therapy shows substantial antidepressant effects that may be durable at least through 12 months following acute intervention in some patients. 75% of participants met the criteria for clinical response (defined as a ≥50% reduction in GRID-HAMD scores), and 58% met the criteria for remission (GRID-HAMD ≤7). These results were accompanied by a large effect size (Cohen’s d = 2.4), indicating a sustained and meaningful antidepressant effect. |
| Daws et al., 2022. | United States Of America and United Kingdom | Two clinical trials: an open-label trial and a double-blind randomized controlled trial (DB-RCT). | Open-label:  N= 16 (4 females)  Mean age = 42.75 years.  Ethnicity = not assessed.  DB-RCT:  N1 = 22 (6 females)  Mean age = 40.9 years  N2 = 21 (8 females)  Mean age = 44.5 years .  Ethnicity = not assessed. | To assess the antidepressant potential of psilocybin and its effects on brain function. | Open-label (2 sessions), DB-RCT (2 sessions of 25 mg psilocybin or 1 mg psilocybin with escitalopram). | Functional magnetic resonance imaging (fMRI). | Psilocybin and escitalopram. | Not related | Psilocybin therapy resulted in decreased brain network modularity, indicating increased global brain network integration. This change correlated with improvements in depression symptoms, suggesting a potential mechanism for the antidepressant effects of psilocybin. |
| Goodwin et al., 2022 | 22 sites across 7 countries in Europe and North America | Random assignment of adults with treatment-resistant depression to receive a single dose of synthetic psilocybin (25 mg, 10 mg, or 1 mg) with psychological support. | N = 233 (52% females)  Mean age = 39.8 years  Ethnicity = 92% were White | To determine the efficacy and safety of a single dose of psilocybin in treating treatment-resistant depression. | One administration session, with follow-up over 12 weeks. | Montgomery–Åsberg Depression Rating Scale (MADRS) | Synthetic formulation of psilocybin (25 mg, 10 mg, or 1 mg) | Headache, nausea, dizziness, and in some cases, suicidal ideation or self-injury. | Psilocybin (25 mg) showed efficacy in reducing depression scores at 3 weeks compared to 1 mg, but with notable adverse effects. The 10 mg dose did not significantly differ from the 1 mg dose. Further studies are needed to evaluate long-term efficacy and safety. |
| Carhart-Harris et al., 2021 | United Kingdom | Double-blind, randomized controlled trial | N = 59 (29 male, 20 female)  Mean age = 41 years  Ethnicity = 52 (85%) participants were White | To explore the impact of therapeutic alliance and rapport on depression outcomes in psilocybin-assisted therapy | Two psilocybin dosing sessions | Quick Inventory of Depressive Symptomatology (QIDS), Scale To Assess the Therapeutic Relationship (STAR-P) | Psilocybin (25 mg) | Not specifically mentioned | Although the study authors failed to show a significant difference between the psilocybin and escitalopram groups at 6 weeks in the designated primary outcome measure, most secondary outcomes, including other depression severity scores, favored the psilocybin group. Notably, the researchers highlighted that the acute subjective effects of psilocybin, such as emotional breakthroughs and mystical-type experiences, were strong predictors of positive clinical outcomes. These experiences were associated with more significant decreases in depression scores. |
| Murphy et al., 2022 | United Kingdom | Phase 2, double-blind, randomized controlled trial | N = 29 (11 females)  Mean Age = 42.8 years  Ethnicity = 27 participants (93%) identified as White | Compare effects of psilocybin and escitalopram in treating depression | Two doses over 6 weeks | Quick Inventory of Depressive Symptomatology–Self-Report (QIDS-SR-16) | Psilocybin and Escitalopram | Similar incidence of adverse events in both groups | Larger and longer trials needed to compare psilocybin with established antidepressants |
| Davis et al., 2021 | United States Of America | Randomized, waiting list–controlled clinical trial | N = 24 (16 women and 8 men)  Mean age = 39.8 years  Ethnicity = not assessed. | Investigate the effect of psilocybin therapy in patients with Major Depressive Disorder (MDD) | Two psilocybin sessions | Structured assessments (e.g., SCID-5, SCID-5 Screening  Personality Questionnaire, SCID-5 Personality Disorders, and Personality Assessment Inventory). | Psilocybin (session 1: 20 mg/70 kg; session 2: 30 mg/70 kg) | Transient increase in blood pressure, challenging emotional experiences, mild to moderate transient headache | Psilocybin-assisted therapy is efficacious in producing large, rapid, and sustained antidepressant effects in patients with MDD |
| Madsen et al., 2020. * | Denmark. | The study involved PET imaging, psychological assessments, and a single oral dose of psilocybin. | N = 10 (4 females)  Mean age = 28.4  Ethnicity = not assessed. | To evaluate the long-term effects of psilocybin on mindfulness and personality, and its relation to 5-HT2A receptor binding. | Single psilocybin session. | [11C]Cimbi-36 PET imaging was used. | Psilocybin (0.2–0.3 mg/kg). | Specific side effects were not detailed in the accessible parts of the document. | A single dose of psilocybin led to long-term increases in mindfulness and openness, without a consistent change in neocortical 5-HT2A receptor binding. |
| Carbonaro et al., 2018 * | United States Of America | A double-blind method was used. | N = 20  (gender not assessed)  Mean age = 28.5 years​.  Ethnicity = 19 were Caucasian (95%) and one was Asian American. | The aim was to directly compare the effects of psilocybin and DXM in the same participants, focusing on alterations in subjective experience​. | There were five experimental sessions, each lasting about 7 hours, and a final follow-up session​. | Instruments included blood pressure cuffs, pupilometers, and various questionnaires like the Subjective Effects Questionnaire, Altered States of Consciousness (5D-ASC), States of Consciousness Questionnaire, Mysticism Scale, Psychological Insight Questionnaire, Challenging Experience Questionnaire, and Hallucinogen Rating Scale​. | The drugs used were psilocybin (10, 20, and 30 mg/70 kg) and dextromethorphan HBr (400 mg/70 kg). | Notable side effects included nausea and emesis, particularly with DXM. No participant vomited after receiving placebo or lower doses of psilocybin, but 55% did after receiving 400 mg/70 kg DXM | The study found that high doses of psilocybin and DXM produced similar overall perceived strengths of drug effects. However, the 30 mg/70 kg dose of psilocybin often produced significantly greater effects than DXM on various subscales and questionnaires assessing mystical experiences, psychological insights, and visual effects. |
| Barrett et al., 2018 * | United States Of America | Double-blind, placebo-controlled, counterbalanced, crossover design | N = 20 (11 females)  Mean Age = 28.5 years  Ethnicity = 19 Caucasian, 1 Asian American | To compare cognitive effects of psilocybin and dextromethorphan (DXM) | 5 sessions per participant, with varying doses of psilocybin and one high dose of DXM​ | Motor Praxis task;Memory;Working Memory and Vigilance;Executive Function and Overall Cognitive Impairment;The Penn Line Orientation Test (PLOT).Computerized Neurocognitive Battery (CNB);his involved the Digit Symbol Substitution Task, measuring executive function, mental flexibility, and associative learning, and the Mini-Mental Status Examination (MMSE). | Psilocybin (low, medium, high doses: 10, 20, 30 mg/70 kg) and DXM (high dose: 400 mg/70 kg) | Drug-induced impairment in cognitive domains; DXM showed greater cognitive impairment compared to psilocybin​ | Psilocybin and DXM differ in their cognitive effects. Psilocybin had greater effects on working memory, whereas DXM had more pronounced effects on episodic memory, response inhibition, and executive control. DXM use poses greater risks if abused​ |

*TRD*: Treatment-resistant depression

* Studies using healthy participants.

*Note: The* ***Montgomery–Åsberg Depression Rating Scale (MADRS)*** *is a clinician-administered tool to assess depressive symptom severity. It focuses on core symptoms such as sadness, tension, reduced sleep, appetite, concentration, and inability to feel. Scores range from 0 to 6 per item, with higher scores indicating greater severity (Montgomery & Åsberg, 1979).*

*The* ***GRID-Hamilton Depression Rating Scale (GRID-HAMD)*** *is a standardized clinician-administered tool for assessing depression severity across mood, sleep, appetite, and physical symptoms. Higher scores reflect more severe depression, while lower scores suggest improvement (Williams, 1988; Williams et al., 2008).*

*The* ***Quick Inventory of Depressive Symptomatology–Self-Report (QIDS-SR-16)*** *evaluates depressive symptoms such as mood, sleep, weight, and energy through patient self-report. Scores indicate depression severity (Rush et al., 2003).*

*The* ***Beck Depression Inventory (BDI)*** *is a self-report tool assessing cognitive, emotional, and physical depression symptoms, with higher scores reflecting greater severity (Beck et al., 1961).*

*The* ***Sheehan Disability Scale (SDS)*** *measures functional impairment across work, social, and family domains, offering insights into how depressive symptoms interfere with daily life (Sheehan, 1983).*

*The* ***Five-Dimensional Altered States of Consciousness Rating Scale (5D-ASC)*** *assesses subjective altered states during psychedelic experiences. It evaluates dimensions such as oceanic boundlessness, visual restructuralization, and ego dissolution (Dittrich, 1998).*

*The* ***Positive and Negative Affect Schedule (PANAS)*** *measures mood states by assessing positive and negative affect dimensions, offering insights into emotional responses during interventions (Watson et al., 1988).*

*The* ***Generalized Anxiety Disorder Scale (GAD-7)*** *is a widely used self-report tool that evaluates the severity of generalized anxiety symptoms, with higher scores indicating greater severity (Spitzer et al., 2006).*

*The* ***Work and Social Adjustment Scale (WSAS)*** *measures functional impairment in work, social, and personal domains, providing an understanding of the impact of mental health conditions on daily life (Mundt et al., 2002).*

*The* ***EQ-5D-3L*** *is a standardized instrument for measuring health-related quality of life. It assesses mobility, self-care, usual activities, pain/discomfort, and anxiety/depression (EuroQol Group, 1990).*

*The* ***Digit Symbol Substitution Test (DSST)*** *measures cognitive functioning, including attention, processing speed, and executive function, often used in clinical studies involving neurological or psychological conditions (Wechsler, 1955).*

*The* ***Adjective Mood Rating Scale (AMRS)*** *evaluates mood changes, including calmness, energy levels, and happiness, and is often used in studies involving psychoactive substances (Janke & Debus, 1978).*

*The* ***Mystical Experience Questionnaire (MEQ)*** *measures mystical or spiritual experiences during interventions like psilocybin. It focuses on feelings of unity, transcendence, and sacredness (Barrett et al., 2015).*

*The* ***Emotional Breakthrough Inventory (EBI)*** *assesses emotional release and transformation, capturing moments of resolving struggles and gaining new perspectives during therapy (Roseman et al., 2019).*

*The* ***Big Five Inventory (BFI)*** *and the* ***Big Five Aspects Scale*** *assess personality traits across five dimensions: openness, conscientiousness, extraversion, agreeableness, and neuroticism (John & Srivastava, 1999).*

*The* ***Modified-Tellegen Absorption Scale*** *measures individuals' openness to immersive and transformative experiences, often in psychedelic contexts (Tellegen & Atkinson, 1974).*

*The* ***Barratt Impulsivity Scale-Brief*** *evaluates impulsivity across cognitive, motor, and non-planning domains, commonly used in psychological studies (Patton et al., 1995).*

*The* ***Ruminative Response Scale (RRS)*** *measures the extent to which individuals focus on negative thoughts and emotions, often used in depression studies (Nolen-Hoeksema, 1991).*

*The* ***White Bear Suppression Inventory (WBSI)*** *assesses thought suppression tendencies, which are associated with mental health outcomes, including depression and anxiety (Wegner & Zanakos, 1994).*

*The* ***Visual Analog Scale (VAS)*** *is a simple tool for measuring subjective experiences, such as mood or pain intensity, by having participants mark a point on a line representing their experience (Huskisson, 1974).*

*The* ***QLES-Q-SF*** *measures the degree of enjoyment and satisfaction individuals experience across various areas of daily life, including physical health, mood, work, and social relationships. It is widely used in psychiatric research and clinical trials to assess quality of life, particularly in depression and related disorders.
 (****Endicott, Nee, Harrison, & Blumenthal, 1993****)*
