# Supplementary material for: Therapeutic Use of Psilocybin in Depression: a Systematic Review of Clinical Evidence
Source: Acta Neuropsychiatr. 2025 Sep 3;37:e86. doi: 10.1017/neu.2025.10039 (PMC13130258; doi:10.1017/neu.2025.10039)
Supplement: Andrade et al. supplementary material [file S0924270825100392sup001.zip › Table 3_ PICO Framework.docx]

**Table 3: PICO Framework**

| **PICO Element** | **Inclusion Criteria** | **Exclusion criteria** |
| --- | --- | --- |
| Participants or population | Adults with depression, MDD, or TRD | Under 18 years or any diagnosis other than depression. |
| Intervention or exposure | Psilocybin treatment or antidepressants | - |
| Comparators or Control (where relevant) | Healthy participants or control conditions will include patients without depression who receive a placebo in the clinical process and comparison with other drugs, such as Ketamine, Niacin, or Mirtazapine. | - |
| Outcomes | Measures of emotional state (i.e., biological and subjective).  Measures of neural activity (i.e., results from EEG, fMRI, or other methods). Measures of subjective interpretation (i.e., preference and familiarity). | - |
| Study design | RTC's | Systematic reviews, meta-analyses, case reports, animal studies, editorials, expert opinions, conference abstracts |

^Abbreviations:^

**^MDD^**^: Major Depressive Disorder;^ **^PICO^**^- P- Participants, I- Intervention, C- Comparators or control, O-Outcomes;^ **^TRD^**^: Treatment Resistant Depression.^

**^EEG^** ^–^ *^Electroencephalography^*^;^ **^fMRI^** ^–^ *^Functional Magnetic Resonance Imaging.^*
